# Domesticating Social Alarm Systems in Nursing Homes: Qualitative Study of Differences in the Perspectives of Assistant Nurses

**DOI:** 10.2196/44692

**Published:** 2023-05-05

**Authors:** Fangyuan Chang, Britt Östlund, Sanna Kuoppamäki

**Affiliations:** 1 Department of Biomedical Engineering and Health Systems, KTH Royal Institute of Technology Stockholm Sweden; 2 Department of Design Shanghai Jiao Tong University Shanghai China

**Keywords:** technology implementation, nursing care, social alarm system, domestication, nursing home, technology integration, long-term care, social alarm, nursing, elder, older adult, aging, gerontology, geriatric, interview, qualitative

## Abstract

**Background:**

New social alarm solutions are viewed as a promising approach to alleviate the global challenge of an aging population and a shortage of care staff. However, the uptake of social alarm systems in nursing homes has proven both complex and difficult. Current studies have recognized the benefits of involving actors such as assistant nurses in advancing these implementations, but the dynamics by which implementations are created and shaped in their daily practices and relations have received less attention.

**Objective:**

Based on domestication theory, this paper aims to identify the differences in the perspectives of assistant nurses when integrating a social alarm system into daily practices.

**Methods:**

We interviewed assistant nurses (n=23) working in nursing homes to understand their perceptions and practices during the uptake of social alarm systems.

**Results:**

During the four domestication phases, assistant nurses were facing different challenges including (1) system conceptualization; (2) spatial employment of social alarm devices; (3) treatment of unexpected issues; and (4) evaluation of inconsistent competence in technology use. Our findings elaborate on how assistant nurses have distinct goals, focus on different facets, and developed diverse coping strategies to facilitate the system domestication in different phases.

**Conclusions:**

Our findings reveal a divide among assistant nurses in terms of domesticating social alarm systems and stress the potential of learning from each other to facilitate the whole process. Further studies could focus on the role of collective practices during different domestication phases to enhance the understanding of technology implementation in the contexts of complex interactions within a group.

## Introduction

### Background

**Figure 1:**
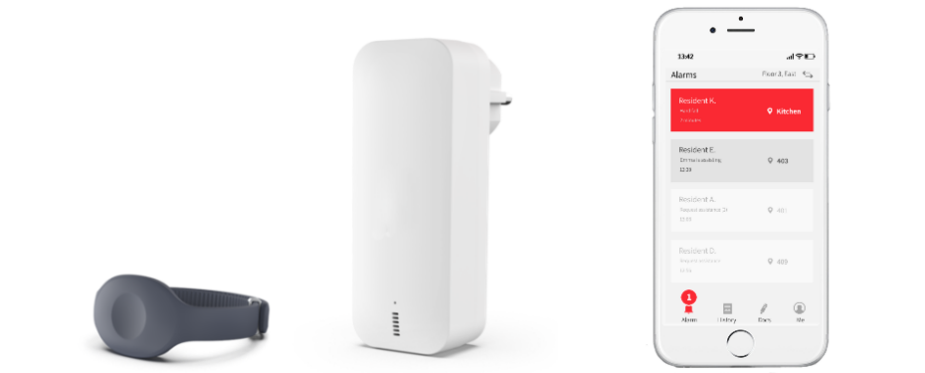
A social alarm system: alarm button (left), base unit (middle), care phone (right).

In Sweden and in many other countries, social alarm systems have been included in public social services [1] to alleviate the global challenge of aging populations [2], compensate for the shortage of skilled care staff [3,4], and increase peace of mind to older adults and their caregivers [5]. The system usually consists of alarm buttons, care phones, and base units (Figure 1). When the system is installed in nursing homes, residents can click alarm buttons for help. Meanwhile, assistant nurses can quickly recognize patient needs or emergencies by checking alarms and talking to patients remotely via their care phones. The system has a range of features including enhancing remote communication between residents and assistant nurses in nursing homes, empowering assistant nurses to call for colleagues’ help when in an emergency, and providing information for care management [6].

Many older social alarm systems require conversion to digital versions for stable signal transfer and advanced functionality. However, the uptake of a new social alarm system into everyday care practices is a key challenge [7,8]. There are often discrepancies between expected and actual implementation outcomes in nursing homes. Most implementation projects do not last beyond pilot periods or only partially deliver on the promise of new social alarm solutions [9]. Users’ concerns about privacy and security may inhibit their system acceptance [10,11]. The expected technology usage patterns may not match the local care practices [12]. In theorizing the failures of technology implementations, scholars note implementers often use the same implementation strategies and expect the same outcomes regardless of the contextual differences [13-15]. The uptake of technology depends not only on technical functions but also on the interaction between users and routines and relationships in the social context in which technology is implemented [16-18]. It is therefore important how technology implementation is dynamically conducted by actors involved in daily practices [19,20].

### Assistant Nurses

Assistant nurses (Undersköterska) are the key actors involved in daily practices in Swedish nursing homes. Different from the registered nurses (Sjuksköterska) with a bachelor’s degree, assistant nurses have an upper secondary training diploma [21]. Although there is no explicit regulation of the division of labor between assistant nurses and registered nurses [22], assistant nurses are typically responsible for hands-on care tasks under the supervision of registered nurses in nursing homes [23]. Hence, assistant nurses are typically the ones working in the frontline, using the social alarm system in everyday care services, and receiving alarms directly from residents in nursing homes [24]. Studies in different fields have shed light on the contribution of assistant nurses to technology implementation. The main concerns include assistant nurses’ perceptions toward specific technology [25,26], their agency and roles in technology implementation [15,27,28], the diversity of interactions between assistant nurses and the selected technology [29,30], decision-making and learning process [12,31], ethical problems that are related to safety or autonomy [32], and the process where assistant nurses assigning meaning to the selected system [33]. These studies provide valuable insights into uncovering the social aspects of appropriating a new technology. Yet, the disparities in the uptake of new technology among assistant nurses have been somehow overlooked, as assistant nurses are often viewed as a whole group.

There are indeed a few studies exploring the differences in practices and perspectives [8,34-36]. For example, Harvey et al [34] explored the experience of different stakeholders in adopting self-management solutions. The findings show that people from different groups meet diverse barriers and facilitators when conducting their work. The findings also suggest that “considering ‘barriers’ and ‘facilitators’ for implementation may be too simplistic,” as the implementation is dynamic where people face different barriers and facilitators throughout the whole process. The research thus highlights that more work is needed to unravel the dynamics of technology implementation. Yet, these studies focus on stakeholders that belong to different groups, rather than people from the same group. That is, there lacks a theoretical and practical understanding of how implementations are created and shaped in practice among assistant nurses.

In this study, we ask how assistant nurses think and act to facilitate the uptake of the social alarm system in practice, and whether and how they have different perceptions and practices during the process. To answer the question, we interviewed 23 assistant nurses in 1 nursing home, captured and critically discussed the dynamics within the group. This study draws on domestication theory, which is widely used to capture and analyze the dynamics within technology implementation processes [37,38].

### Technology Domestication

Against a deterministic discourse that views technology’s meaning and actual use as predictable outside a social context, domestication theory claims—on good empirical grounds—that technology’s meaning and use are the results of interaction with the social context where it is implemented. The theory is especially potent in investigating how users relate to and interact with technology, in practice, over time [19]. Although current domestication studies have paid significant attention to technology use in home settings [37,38], scholars believe the theory can also be applied in workplaces, like institutions of higher education [39]. Using domestication theory in this study can help reveal the complexities of implementing social alarm systems into nursing homes from a practical perspective. Hence, we draw on the theory for exploring how assistant nurses organize their work when implementing social alarm systems in nursing homes.

Originally developed in home settings studies, domestication theory outlines 4 phases of technology implementation: appropriation, objectification, incorporation, and conversion [40-42]. Appropriation refers to the phase where individuals initially encounter new technology. Key themes here focus on possession and ownership. Hence, questions during the process are about how individuals decide to obtain (or not to obtain) new technology and related services based on interpretations of a variety of resources (eg, social resources and material resources). However, in this study, early encounters between the social alarm system and assistant nurses are in working contexts where assistant nurses have no power to affect the system procurement. Hence, ownership of the system may differ from that in home settings, as confusion regarding “why it is this system?” or “how the system should be used?” may emerge. Objectification refers to the phase when technology is located spatially and physically. Related questions are therefore what the location of technology should be and how to locate it. In our case, it represents how the social alarm system becomes a physical object in nursing homes. This includes examples such as the distribution and installation of social alarm devices. Incorporation refers to the phase involving the temporal organization of technologies. Slightly different from objectification, which concerns the physical integration of technology, incorporation pays attention to how people or contexts shape and reshape technology by organizing it into everyday temporal structures. Hence, combined with the purpose of this study, questions here are about how often, by whom, and when the social alarm system should be used. Conversion refers to the phase where attention is focused on “how people mobilize these technologies as part of their identities and how they present themselves to others” [43]. Key issues in this phase regard how people conduct technology-mediated practices and how they display these practices to others.

Following insights from domestication theory, the implementation of the social alarm system should be viewed as an open and interactive process where the system is integrated into, and as a part of, daily structures. During the process, involved actors face a variety of changes in relation to divergent activities [44], spaces [16], times [45], and goals [17]. The use of the social alarm system is embedded in daily care practices and varies according to specific spatial and social situations in nursing homes. In this sense, viewing the system’s implementation through the lens of domestication theory entails a practice viewpoint that centers on the actual implementation work of assistant nurses.

## Methods

### Data Collection

The study included 1 nursing home in Sweden. By contacting development specialists across Swedish municipalities, we made a preliminary selection of nursing homes. To be included, nursing homes should be implementing or have just implemented a social alarm system within the past 6 months. Although many nursing home leaders were interested in the research, few of them met the inclusion criteria. Three nursing homes were selected. We then distributed questionnaires to employees in the selected nursing homes to screen the nursing homes that were interested in the study. There was only 1 nursing home where most employees agreed with the research. As a result, only 1 nursing home was selected.

Twenty-three assistant nurses were included in the study (Multimedia Appendix 1). Each individual gave formal consent. To recruit assistant nurses, we distributed questionnaires in which assistant nurses could express their desire to participate in interviews. We first contacted those who expressed interest and then used snowball sampling to find other assistant nurses [46]. As a result, we interviewed 23 assistant nurses between October 2019 and December 2020. Of the 23 interviews, 21 were in-person interviews, while 2 were web-based interviews. The in-person interviews took place in private offices and lasted about 60 minutes. The web-based interviews were carried out through Zoom (Zoom Video Communications Inc) when the interviewees were in a comfortable environment (eg, private offices or homes). Each web-based interview lasted about 40 minutes. Only interviewees’ voice was recorded. Saturation was used as a stopping criterion as data collection ended when for 3 consecutive interviews with no major new insights emerged [47]. Interviews were taped and transcribed.

The interviews were semistructured. They evolved around questions regarding the domestication process, as well as assistant nurses’ work in general [48]. The questions include what they do, the challenges they face, how they cope with these challenges in domesticating the social alarm system, and why they work in their described ways (Multimedia Appendix 2). To gain their insights more accurately and completely, we invited interviewees to provide specific examples, and, if possible, to compare the implementation project in their current nursing homes with that in other nursing homes. We also encouraged them to think about whether, how, and why they would change certain work habits if experiencing the current implementation project again [49,50]. The interviews are used to capture interviewees’ detailed thoughts, feelings, and practices when they are domesticating the social alarm system in everyday work.

In addition, we also observed care work before and during the study. Through accompanying the assistant nurses, we aim to achieve an in-depth understanding of the problems and situations mentioned by the interviewees, thereby enabling a full picture of technology domestication in practice [51]. In our case, the observation is only used for helping authors gain an accurate understanding of the interviewees’ experience with the domestication of social alarm systems in nursing homes. Hence, no observation data were included in the data analysis.

### Data Analysis

The transcripts were analyzed in NVivo (version 12; QSR International) through an abductive form of content analysis [52,53]. As the study aims to identify the differences in assistant nurses’ domestication of a social alarm system, the first author categorized the collected data into 4 groups according to their association with the 4 domestication phases: the group of appropriation phase data, the group of objectification phase data, the group of incorporation phase data, and the group of conversion phase data. Within each group, the first author coded the transcripts in an attempt of understanding how assistant nurses think and act in different domestication phases. This included line-by-line reviewing transcripts, extracting relevant text segments, and identifying codes (n=18), with a focus on what assistant nurses tried to do and how they did it. Next, the subthemes (n=8) related to participants’ different approaches to domesticating the system in each phase were identified. The codes and subthemes were then jointly discussed by the authors, and further refined into broader themes (n=4) about the differences among assistant nurses under the 4 domestication phases. Examples of data extraction are listed in Multimedia Appendix 3. This tactic empowered us to identify the different ways of understanding and conducting domestication practices and processes among assistant nurses (Table 1). To ensure the validity of the codes, member checking was used as the authors contacted the participants to verify the interpretations of the data [54]. Specifically, the synthesized results about assistant nurses’ domestication of the system were provided to the participants to check for resonance and accuracy with their experiences.

**Table 1:** The assistant nurses’ approaches to domesticating the system in different phases.

Themes (n=4): Differences under specific phases, and subthemes (n=8): participants’ different approaches			Codes (n=18): Participants’ actions and perceptions
**System conceptualization** **(** **appropriation phase** **)**			
	External searching	Search for information from external channels Develop a systematic view	
	Local searching	Learn about the system by using it in reality Develop a realistic view	
**Spatial employment of social alarm devices** **(** **objectification phase** **)**			
	Modular approaches	Categorize the work into clusters with distinct goals Manage the clusters linearly Predefine metrics to evaluate the work	
	Hands-on approaches	Decide the next actions as the implementation proceeds Dynamically organizing the implementation tasks Evaluate the tasks through situated analysis	
**Treatment of unexpected issues** **(** **incorporation phase** **)**			
	Eliminate uncertainties	Trace fundamental reasons for unexpected issues Avoid meeting similar issues again	
	Manage uncertainties	Develop immediate solutions Accept potential unexpected situations	
**Technology literacy promotion** **(conversion phase)**			
	Standardization	Promote individual technology literacy Regulate the procedure of system use	
	Localization	Improve the team cooperation ability and cohesion Create collaborative strategies of technology use	

### Ethical Approval

According to Swedish law, this study does not require an ethics review as it does not collect participants’ personal information, biological materials, and does not have any physical or mental effects on participants [55]. The study has been performed in accordance with the Declaration of Helsinki. All participants gave both verbal and written informed consent. Participants were informed that they could withdraw their consent at any time. Additionally, all participants were given anonymous identifiers to ensure personal privacy during data analysis. Although this study aims to understand the work of assistant nurses, indirect references to the residents in nursing homes were unavoidable. To ensure the confidentiality of residents, we recorded no identifiable information.

## Results

### Appropriation Phase: Differences in System Conceptualization

#### Overview

In the appropriation phase, assistant nurses were not involved in making purchase decisions but directly used the system after the nursing home leader finished the procurement process. Consequently, unlike users in households who pay close attention to which technology to procure, assistant nurses mainly considered the following: “why did we choose this system?” and “what are the benefits of the system?”

When encountering the new system, assistant nurses used different approaches to interpreting the system’s aims, objectives, and expected benefits. In the following subsections, we show how the assistant nurses figured out the system’s purposes and benefits differently.

#### External Searching

We do great searching work … the official website shows the system’s benefits… We also learn from those who use it in other places. …If you want your system to be used quickly and smoothly, you need to know all the key information, so that you have a systematic view…to acquire the best paradigms of choice.

AN01

This quote illustrates how assistant nurses used external channels (eg, the internet and other users) to broadly identify the value, benefits, and importance of using the system. They linked the collected information to develop a systematic understanding of the newly introduced system, which they believed would contribute to its quick and smooth use in different care contexts.

#### Local Searching

Some assistant nurses emphasized the dynamics of technology benefits by exemplifying how the same function may bring opposite impacts on care practices. These assistant nurses indicated that they preferred conceptualizing the system by actually using it in daily work. In doing so, they developed a realistic understanding of technology concepts under local contexts, which, in their mind, allowed them to face different local challenges.

Things always change, and I prefer to use it and feel it (instead of searching for online information) to overcome daily challenges in local...For example, it is claimed that the calling function enables everyone peace of mind. But very often I feel stressed, especially if I receive a sudden alarm when caring for one resident...If I stop the ongoing task, I might irritate the resident. If I continue my work, I might miss emergencies. It is not always peaceful.

AN05

### Objectification Phase: Differences in Spatial Employment of Social Alarm Devices

#### Overview

In the objectification phase, assistant nurses, as experts using technology to care for residents, had strong agency in deciding the spatial employment of social alarm devices. Their work in this phase included, for instance, system installation, internet connection, and device distribution. Consequently, they may need to allocate divergent resources (eg, charging stations, alarm phones, transmitters, and sensors) and negotiate with various stakeholders (eg, registered nurses, colleagues, residents, IT support, and installation personnel).

It was found that the assistant nurses developed different approaches to organizing social alarm devices and related resources. In the following subsections, we show how assistant nurses conducted their work differently.

#### Modular Approaches

Some assistant nurses categorized their work into clusters with distinct goals, managed these clusters one by one, and evaluated task completion via predefined metrics. One assistant nurse explained:

...I categorize my work into different clusters…for example, distributing those devices…I count the specific number of different devices, record how many units have received which device, and explore if and how many additional resources are needed…I then move on to another cluster when my work here is done.

AN03

In this case, the device implementation process was seen as linear, within which all implementation work was separated and analytically separable. By using this approach, the implementation progress for the target residents was consistent (eg, the target residents can receive social alarm devices and get trained at the same time).

#### Hands-on Approaches

One assistant nurse emphasized her very open and practical mindset when spatially organizing related devices:

I put the social alarm pendants beside their (residents’) pillows... You can see how they look and act when activating the pendant…Then you know if you need to help them…and how to help them...or if you need to relocate the transmitters...We think about things as they proceed, we try different approaches during the process to know how the system can be better integrated into our work.

AN12

Unlike the assistant nurses above, who had a structural and modular plan, the assistant nurses here gained ideas about what to do next while engaging in device organization and interacting with their residents. They dynamically organized related devices on the basis of the target residents’ feedback and evaluated the implementation progress through situated analysis. Thus, the device implementation process was seen as dynamic, within which all implementation work was defined according to specific contexts. By using this approach, the target residents may receive different implementation solutions and have inconsistent implementation progress.

### Incorporation Phase: Differences in the Treatment of Unexpected Issues

#### Overview

In the incorporation phase, the assistant nurses began to use the social alarm system for daily care services in practice. All participants paid intensive attention to how and when to embed the system into everyday temporal structures. However, many unexpected issues emerged and disrupted the implementation progress during this phase, such as the system having functional errors for unknown reasons and residents unintentionally destroying their alarm buttons. These uncertainties prevented assistant nurses from driving their incorporation work.

Our data reveal 2 different perspectives of assistant nurses on viewing and dealing with uncertainties: eliminating and managing. The following subsections elaborated on the differences.

#### Eliminating Uncertainties

I watch and ask, and look into the situation here and now, to understand what exactly caused the unexpected issue, and in what way…I try to make the issues disappear all at once.

AN06

Many of the assistant nurses aimed to eliminate uncertainties by learning from unexpected issues and taking related precautions. They tended to determine the fundamental reasons behind the issue, based on which they developed corresponding coping strategies that could prevent similar issues in the future.

#### Managing Uncertainties

Instead of addressing unexpected issues from the root, some assistant nurses chose to manage uncertainties:

There are always unexpected situations...no need to spend time and energy killing the uncertainties…What is important is to get things done. If the phone doesn’t work, change it; if there are many false alarms, fine, let’s regularly check our residents…find the immediate solutions, (and) let the residents get cared.

AN01

These assistant nurses strongly believed that unexpected problems were unavoidable. They emphasized that, compared to eliminating uncertainties, finding immediate and alternative solutions to accomplish tasks, getting things done, and caring for the residents were of the highest importance.

### Conversion Phase: Differences in Technology Literacy Promotion

#### Overview

In the conversion phase, the assistant nurses focused on how to construct their technology use practices. However, due to the different levels of technology literacy, inconsistent technology use practices within the group were found. Most assistant nurses believed that this might cause risks to the health or safety of residents; thus, relevant actions were needed.

However, the participants held different opinions on which actions should be taken to deal with uneven technology literacy. The following subsections show how assistant nurses had different attitudes toward technology literacy promotion.

#### Standardization

Some assistant nurses worked on standardizing the technology use, by promoting individual technology literacy and regulating the procedures of system use (eg, using standardized terms and work routines). The following assistant nurse explained how he created uniform rules of conduct for recharging social alarm devices:

I regulate the procedure of charging alarm phones in our ward…It helps to make everything in order and efficient as all of us (assistant nurses) know what to do…I explain to my colleagues who don’t know. We have to spare time to deal with these problems.

AN02

This quote highlights how these assistant nurses viewed the inconsistency in technology use as a personal issue. They set regulations for assistant nurses’ practices and work processes. By educating colleagues with less technology literacy, they believed that all assistant nurses could make “everything in order and efficient.”

#### Localization

Other assistant nurses claimed that the inconsistent technology use across individuals could still lead to high-quality care if people localize the system use in their teams:

…what matters is how to use the system to benefit our care provision…we work as a team, and I can thus support my colleagues if they have difficulties in using the system…we should recognize this is a team. So my colleagues and I improved our collaboration strategies, and we use the new system well (even though we have different levels of technology literacy).

AN17

In this case, the assistant nurse argued that assistant nurses with less technology literacy can still use the system well, as they can receive support from other team members. Consequently, instead of promoting individual technology literacy, they worked on promoting team cooperation and cohesion, as well as creating collaborative strategies for technology use.

## Discussion

### Principal Findings

When it comes to domesticating the system in practice, we found a divide within the group of assistant nurses. Drawing on domestication theory, we identified 4 differences among assistant nurses, including conceptualizing the system, spatially organizing the system, treating unexpected issues, and promoting technology literacy. During different domestication phases, assistant nurses developed multiple strategies to locate the system in the “right place” under given contexts.

### A Divide Within the Group of Assistant Nurses

Zooming in on the assistant nurses’ strategies, we found that some assistant nurses “sink down” to focus on the actual functioning of the system in working environments, while some assistant nurses “float up,” focusing on a broader arrangement of strategies in the implementation project.

The finding enables a more comprehensive understanding of how technology is integrated into everyday care services. The idea that people have differences in technology implementation is not new [34,45,56,57]. Gjestsen et al [57], for example, pointed out that it is challenging to align stakeholders’ interests when planning technology integration, as there are multiple influential factors to consider at different organizational levels. Similarly, Harvey et al [34] identified different barriers and facilitators that stakeholders meet in an implementation project. In addition to the findings, we unravel the divide between people who are in the same expert group, showing that differences do not only exist across groups but also within groups.

Besides, we show the differences in the same group should be considered from a practice perspective. Against the backdrop that reality is not something pre-existing and stable, but constantly made in practice [45,56,57], we show how the differences between assistant nurses emerge and are handled under different domestication phases in practice. Our findings capture a theme underscoring that differences between assistant nurses are situated and contextual as they emerge in different domestication phases. We argue studies about differences need to consider the dynamics within a group and take particular contexts or phases into account. This is in line with current knowledge that technology should not be viewed as value-neutral [37] and the whole process should be seen as dynamic [8,12].

### The Potential of Compensating Group Members in Technology Domestication

The findings also show that assistant nurses can compensate their group members on the other side in terms of technology domestication. During the appropriation phase, if the system’s concepts, as derived from external searching, can combine with those concepts derived from local searching, the assistant nurses would be clear about the system’s benefits and shortcomings in general and under particular care contexts. During the objectification phase, some assistant nurses continuously bring in the answers of “how things are going” by exploring multiple interactions and connections in the working environments through hands-on approaches. Meanwhile, some assistant nurses make modular plans to ensure the accomplishment of implementation tasks one by one. If these assistant nurses can combine their modular approaches with their colleagues’ hands-on findings, they can reconsider and refine their selection of measurable properties in their modular work. The potential of compensating each other could also be observed in the phases of incorporation and conversion. Through within-group collaborations, these assistant nurses can compensate each other to answer questions regarding “how and where to locate the social alarm system for whom, and to what purposes.” A detailed but systematic landscape regarding system use in practice arises, that otherwise cannot come into being if either side works alone.

In this case, we argue, to overcome identified differences and to efficiently domesticate the system into daily work, assistant nurses need to build common ground for sharing compensatory knowledge to ensure a shared understanding of key details in technology domestication. Hence, in addition to the studies about involving assistant nurses in implementation management [35,58,59], we call for more studies that shed light on the collective practices during technology domestication, so as to contribute to the development of a common ground for knowledge sharing.

### Limitations

There are several limitations in the collected data, considering the generalizability of our results. First, we recruited and interviewed individuals who expressed interest in the study. Hence, our data cannot explain any relationship between the differences we found and demographics such as age, gender, and care experience. Second, we did not interview nursing home leaders or technology developers who play crucial roles in social alarm system procurement, although their insights would certainly have additional contributions. However, our data do reflect the understanding of assistant nurses regarding their experiences in domesticating the social alarm system, as well as the rationales for their actions and approaches during all domestication phases.

### Conclusions

Our study identified a divide between the assistant nurses who use the system for care provision. The group of people dealt with the complexities related to the system domestication differently as they have distinct goals, focus on different facets, and developed diverse coping strategies. Based on the differences we identified, we highlight the importance of understanding the dynamics in practices when domesticating technology within the same expert group.

## Appendix

Multimedia Appendix 1 Demographics of included assistant nurses.

Multimedia Appendix 2 Interview Guide.

Multimedia Appendix 3 Examples of data extraction.

## References

[1] (2021). Ramavtal för upphandling av trygghetslarm. Sveriges Kommuner och Regioner.

[2] United Nations (2020). World Population Ageing 2020 Highlights.

[3] Marć M, Bartosiewicz A, Burzyńska J, Chmiel Z, Januszewicz P (2019). A nursing shortage: a prospect of global and local policies. Int Nurs Rev.

[4] (2021). Global strategic directions for nursing and midwifery. World Health Organization.

[5] De San Miguel K, Lewin G, Burton EL, Howat P, Boldy D, Toye C (2017). Personal emergency alarms: do health outcomes differ for purchasers and nonpurchasers?. Home Health Care Serv Q.

[6] Stokke R (2017). "Maybe we should talk about it anyway": a qualitative study of understanding expectations and use of an established technology innovation in caring practices. BMC Health Serv Res.

[7] Pritchard GW, Brittain K (2015). Alarm pendants and the technological shaping of older people's care. Technol Forecast Soc Change.

[8] Murray E, Burns J, May C, Finch T, O'Donnell C, Wallace P, Mair F (2011). Why is it difficult to implement e-health initiatives? A qualitative study. Implement Sci.

[9] (2021). Ehälsa och välfärdsteknik i kommunerna. Socialstyrelsen.

[10] Stokke R (2018). Older people negotiating independence and safety in everyday life using technology: qualitative study. J Med Internet Res.

[11] Sjölinder M, Avatare Nöu AA (2014). Indoor and outdoor social alarms: understanding users' perspectives. JMIR Mhealth Uhealth.

[12] Chang F, Kuoppamäki S, Östlund B (2022). Technology scripts in care practice: a case study of assistant nurses' use of a social alarm system in Swedish nursing homes. Digit Health.

[13] Andreassen HK, Kjekshus LE, Tjora A (2015). Survival of the project: a case study of ICT innovation in health care. Soc Sci Med.

[14] Stokke R (2016). The personal emergency response system as a technology innovation in primary health care services: an integrative review. J Med Internet Res.

[15] Chang F, Eriksson A, Östlund B (2020). Discrepancies between expected and actual implementation: the process evaluation of PERS integration in nursing homes. Int J Environ Res Public Health.

[16] Oudshoorn N (2012). How places matter: telecare technologies and the changing spatial dimensions of healthcare. Soc Stud Sci.

[17] Kellogg KC (2022). Local adaptation without work intensification: experimentalist governance of digital technology for mutually beneficial role reconfiguration in organizations. Organ Sci.

[18] Milligan C, Roberts C, Mort M (2011). Telecare and older people: who cares where?. Soc Sci Med.

[19] Oudshoorn N, Pinch T (2003). How Users Matter: The Co-Construction of Users and Technology (Inside Technology).

[20] Introna LD, Hayes N (2011). On sociomaterial imbrications: what plagiarism detection systems reveal and why it matters. Inform Organ.

[21] Fahlström G (1999). Ytterst i organisationen: om undersköterskor, vård-och sjukvårdsbiträden i äldreomsorg (Doctoral dissertation, Acta Universitatis Upsaliensis).

[22] (2021). About the Swedish healthcare system. National Board of Health and Welfare.

[23] Holmberg B, Hellström I, Österlind J (2019). End-of-life care in a nursing home: assistant nurses' perspectives. Nurs Ethics.

[24] (2013). Psykisk sjukdom bland äldre och behandling inom vården (Mental illness among elderly and treatment within health care). The National Board of Health and Welfare.

[25] Bail K, Gibson D, Acharya P, Blackburn J, Kaak V, Kozlovskaia M, Turner M, Redley B (2022). Using health information technology in residential aged care homes: an integrative review to identify service and quality outcomes. Int J Med Inform.

[26] Dugstad J, Sundling V, Nilsen ER, Eide H (2020). Nursing staff's evaluation of facilitators and barriers during implementation of wireless nurse call systems in residential care facilities. A cross-sectional study. BMC Health Serv Res.

[27] Fraczkowski D, Matson J, Lopez KD (2020). Nurse workarounds in the electronic health record: an integrative review. J Am Med Inform Assoc.

[28] Gillam J, Davies N, Aworinde J, Yorganci E, Anderson JE, Evans C (2022). Implementation of eHealth to support assessment and decision-making for residents with dementia in long-term care: systematic review. J Med Internet Res.

[29] Jung MM, van der Leij L, Kelders SM (2017). An exploration of the benefits of an animallike robot companion with more advanced touch interaction capabilities for dementia care. Front ICT.

[30] Chang WL, Šabanović S (2015). Interaction expands function: social shaping of the therapeutic robot PARO in a nursing home.

[31] Qian S, Yu P, Bhattacherjee A (2019). Contradictions in information technology mediated work in long-term care: an activity theoretic ethnographic study. Int J Nurs Stud.

[32] Cuesta M, German Millberg L, Karlsson S, Arvidsson S (2020). Welfare technology, ethics and well-being a qualitative study about the implementation of welfare technology within areas of social services in a Swedish municipality. Int J Qual Stud Health Well-being.

[33] Saborowski M, Kollak I (2015). “How do you care for technology?”: care professionals' experiences with assistive technology in care of the elderly. Technol Forecast Soc Change.

[34] Harvey J, Dopson S, McManus RJ, Powell J (2015). Factors influencing the adoption of self-management solutions: an interpretive synthesis of the literature on stakeholder experiences. Implement Sci.

[35] Nilsen ER, Dugstad J, Eide H, Gullslett MK, Eide T (2016). Exploring resistance to implementation of welfare technology in municipal healthcare services: a longitudinal case study. BMC Health Serv Res.

[36] Peek STM, Wouters EJ, Luijkx KG, Vrijhoef HJ (2016). What it takes to successfully implement technology for aging in place: focus groups with stakeholders. J Med Internet Res.

[37] Aceros JC, Pols J, Domènech M (2015). Where is grandma? Home telecare, good aging and the domestication of later life. Technol Forecast Soc Change.

[38] Brause SR, Blank G (2020). Externalized domestication: smart speaker assistants, networks and domestication theory. Inf Commun Soc.

[39] Habib L (1998). Domesticating learning technologies in a higher education institution: a tale of two virtual learning environments. New Teach Learn Practice: Exp elearning Projects Oslo University College.

[40] Hirsch E, Silverstone R (1994). Consuming Technologies.

[41] Silverstone R (2005). Domestication in Media Technologies.

[42] Silverstone R, Haddon L, Robin M, Silverstone R (1996). Design and the domestication of information and communication technologies: technical change and everyday life. Communication by Design: The Politics of Information and Communication Technologies.

[43] Haddon L, Thorhauge AM, Valthysson B (2018). The domestication of complex media repertoires. The Media and the Mundane: Communication Across Media in Everyday Life.

[44] Bechky BA (2003). Sharing meaning across occupational communities: the transformation of understanding on a production floor. Organ Sci.

[45] Mol A (2008). The Logic of Care Health and the Problem of Patient Choice.

[46] Biernacki P, Waldorf D (1981). Snowball sampling: problems and techniques of chain referral sampling. Sociol Methods Res.

[47] Flick U (2013). The SAGE Handbook of Qualitative Data Analysis.

[48] Foddy W, Foddy WH (1993). Constructing Questions for Interviews and Questionnaires:Theory and Practice in Social Research.

[49] Denzin NK, Lincoln YS (2011). The Sage Handbook of Qualitative Research.

[50] Opdenakker R (2006). Advantages and disadvantages of four interview techniques in qualitative research. Forum Qualitative Sozialforschung Forum: Qualitative Social Research.

[51] Thurmond VA (2001). The point of triangulation. J Nurs Scholarsh.

[52] Timmermans S, Tavory I (2012). Theory construction in qualitative research: from grounded theory to abductive analysis. Sociol Theor.

[53] Graneheim UH, Lindgren B, Lundman B (2017). Methodological challenges in qualitative content analysis: a discussion paper. Nurse Educ Today.

[54] Carter N, Bryant-Lukosius D, DiCenso A, Blythe J, Neville AJ (2014). The use of triangulation in qualitative research. Oncol Nurs Forum.

[55] (2003). The act concerning the ethical review of research involving humans (2003:460). Ministry/authority: The Ministry of Education and Cultural Affairs.

[56] Mol A, Moser I, Pols J (2010). Care: putting practice into theory. Care in Practice.

[57] Gjestsen MT, Wiig S, Testad I (2017). What are the key contextual factors when preparing for successful implementation of assistive living technology in primary elderly care? A case study from Norway. BMJ Open.

[58] Ferlie E, Crilly T, Jashapara A, Peckham A (2012). Knowledge mobilisation in healthcare: a critical review of health sector and generic management literature. Soc Sci Med.

[59] Coeckelbergh M (2013). E-care as craftsmanship: virtuous work, skilled engagement, and information technology in health care. Med Health Care Philos.

