# Supplementary material for: Domesticating Social Alarm Systems in Nursing Homes: Qualitative Study of Differences in the Perspectives of Assistant Nurses
Source: J Med Internet Res. 2023 May 5;25:e44692. doi: 10.2196/44692 (PMC10199381; doi:10.2196/44692)
Supplement: Multimedia Appendix 1 [file jmir_v25i1e44692_app1.docx]

## Appendix 1 Demographics of included assistant nurses

| **Label** | **Age** | **Gender** | **Years of care experience** |
| --- | --- | --- | --- |
| AN01 | 37 | Female | 1 |
| AN02 | 41 | Male | 6 |
| AN03 | 38 | Male | 3 |
| AN04 | 36 | Female | 3 |
| AN05 | 36 | Female | 1 |
| AN06 | 32 | Male | ＜1 |
| AN07 | 45 | Female | 8 |
| AN08 | 44 | Female | 5 |
| AN09 | 39 | Female | 4 |
| AN10 | 39 | Female | 4 |
| AN11 | 33 | Female | 2 |
| AN12 | 45 | Female | 9 |
| AN13 | 49 | Female | 12 |
| AN14 | 47 | Female | 10 |
| AN15 | 47 | Female | 8 |
| AN16 | 46 | Female | 10 |
| AN17 | 38 | Female | 6 |
| AN18 | 43 | Male | 9 |
| AN19 | 35 | Female | 1 |
| AN20 | 33 | Female | 1 |
| AN21 | 39 | Female | 5 |
| AN22 | 44 | Female | 4 |
| AN23 | 47 | Female | 12 |
