# Supplementary material for: Domesticating Social Alarm Systems in Nursing Homes: Qualitative Study of Differences in the Perspectives of Assistant Nurses
Source: J Med Internet Res. 2023 May 5;25:e44692. doi: 10.2196/44692 (PMC10199381; doi:10.2196/44692)
Supplement: Multimedia Appendix 2 [file jmir_v25i1e44692_app2.docx]

## Appendix 2 Interview Guide

| **Focus area** | **Examples of questions and probes** |
| --- | --- |
| Daily practices | - Tell me a little about your day in this nursing home. How do you use the system?  - How was your day here before having the system?  - Have there been changes in your use of the system (the first time using the system VS. now)? Why? |
| Challenges | - How well do you think that you have been supported by using the system?  - Could you please tell me more about the challenges you have met? How did you deal with it?  - Has there been an occasion when something you expected to happen by using the system didn’t happen? Tell me more about that. |
| Future expectation | - Whether you would change certain work habits if experiencing the current implementation project again? If yes, how would you change, why? |
| Note: Questions could be asked in a random order to insure the fluency of the interviews. | |
