# Supplementary material for: Domesticating Social Alarm Systems in Nursing Homes: Qualitative Study of Differences in the Perspectives of Assistant Nurses
Source: J Med Internet Res. 2023 May 5;25:e44692. doi: 10.2196/44692 (PMC10199381; doi:10.2196/44692)
Supplement: Multimedia Appendix 3 [file jmir_v25i1e44692_app3.docx]

## Appendix 3 Examples of data extraction

| Themes | Sub-themes | Codes | Condensed meaningful segments | Meaningful text segments |
| --- | --- | --- | --- | --- |
| System conceptualization (Appropriation phase) | External searching | Search information from external channels | Use external channels (e.g. website, other users) to conceptualize the system | The learning sessions and materials do not mention why the system is good, why we should use it.  No, I didn’t learn (the system benefits) from the training session, I learn it from my colleagues.  We do great searching work......the official website shows the system’s benefits... We also learn from those who use it in other places. |
|  |  | Emphasize the systematic view | Believe the systematic view of the system concepts contribute to quick and smooth technology use | If you want your system to be used quickly and smoothly, you need to know all the key information  you have a systematic view...it helps acquire the best paradigms of choice. |
|  | Local searching | Learn about the system by using it in reality | Eventually conceptualize the system through using, and feeling it in real care practices | Things always change, and I prefer use it and feel it (instead of searching for online information)...  We are learning by using...  I know its potential, but would know its real benefits only by using it in real care practices |
|  |  | Emphasize the realistic view | Showing the conflicts between the expected technology usage and the real technology usage | For example, it is claimed that the calling function enables everyone peace of mind. But very often I feel stressful, especially if I receive a sudden alarm when caring for one resident...If I stop the ongoing task, I might irritate the resident. If I continue my work, I might miss emergencies. It is not always peaceful. |
| Spatial employment of social alarm devices (Objectification phase) | Modular approaches | Categorize the work into different clusters | Split daily tasks into different parts or clusters by listing the daily tasks | ...I categorize my work into different clusters…for example, distributing those devices...  I prefer doing my work orderly. I usually list all my tasks on one piece of paper, and finish them one by one |
|  |  | Manage the clusters one by one | Finish the work that has been categorized into different clusters orderly | ...I then move on to another cluster when my work here is done.  ...finish them one by one |
|  |  | Predetermine metrics to evaluate the work | Evaluate daily work by setting evaluative metrics | I count the specific number of different devices, record how many units have received which device, and explore if and how many additional resources are needed...  I evaluate my weekly work to keep myself clear about the status of task completion |
|  | Hands-on approaches | Highlight the limitations of predetermined metrics | Do not predetermine the metrics for evaluating daily work, as they believe some tasks cannot be evaluated quantitatively | I don’t like setting a goal, like, I should teach five residents about how to use the system today. You cannot guarantee everything works here.  I have a general idea about today’s work, but do not evaluate myself in terms of task completion. It is difficult to evaluate your work about caring. |
|  |  | Try out different ways of system use in reality | Observe the dynamics in local, and use personal ways to employ the system in reality (e.g. teaching older adults about the system use) | I put the social alarm pendants beside their (residents’) pillows... You can see how they look and act when activating the pendant…Then you know if you need to help them...  I would be happy to help them when they meet challenges in using it. You know, sometimes people get annoyed if you keep teaching them something they already know. |
|  |  | Evaluate the process as it proceeds | Think about how to finish and evaluate the task as they proceed | We think about things as they proceed, we try different approaches during the process to know how the system can be better integrated into our work. |
| Treatment of unexpected issues (Incorporation phase) | Eliminate uncertainties | Trace fundamental reasons to unexpected issues | Count the tracing of fundamental reasons to unexpected issues as personal responsibility (e.g. ask technology company, employees) | When we face unexpercted issues, it is my responsibility to know what happened, and why.  There were many technical errors, usually I watch and ask, and look into the situation here and now, to understand what exactly caused the unexpected issue, and in what way.. |
|  |  | Avoid the potentials of meeting similar issues again | See the unexpected situations as issues, and try to overcome the issues all at once even if some tasks are delayed | Some technical issues pose big challenges to our residents, so I try my best to avoid them to happen again. It is worthwhile even though sometimes the tasks need to be delayed.  This has occupied too much of my time and attention...So I try to make the issues disappear all at once. |
|  | Negotiating uncertainties | Develop alternative solutions | Develop immediate solutions to unexpected situations in local so as to get things done | If we can’t handle the uncertainties when using the system, we won’t go this far in this journey. We are here, that means we do well in surviving together with the uncertainties. There is no need to spend time and energy killing the uncertainties.  We cannot hear the alarms, then let’s carry the phone all the time. This device broke out, then let’s use a new one. We need to get things done. |
|  |  | Accept potential unexpected situations | Treat unexpected situations as common | It is very common that we meet unexpected situations, sometimes it is about the system, sometimes it is about the residents...it is not unusual  There are always unexpected situations, and there are always solutions... |
| Technology literacy promotion (Conversion phase) | Standardization | Regulate the procedure of system use | Make everything in order by regulating the procedure of technology use | I regulate the procedure of recharging alarm phones in our ward...It helps to make everything in order and efficient as all of us (assistant nurses) know what to do... |
|  |  | Establish the same training materials | Add details to existing training materials to ensure that employees use the system in the same way | Though many training materials are not that sufficient, but by adding more details in it, we can learn the same knowledge, and use the system in the same way. |
|  |  | Work on increase staff’s ability of system use | Set the goal of ensuring the same level of technology literacy among employees | ...we can learn the same knowledge, and use the system in the same way. |
|  |  | View the system use as an individual event | Treat the inconsistency of technology as problematic, and teach the assistant nurses who have low technology literacy | I explain to my colleagues who don’t know. We have to spare time to deal with these problems. |
|  | Localization | Work on ensuring patient safety and satisfaction | Break the expected ways of technology use to ensure patient safety and satisfaction | what matters is how to use the system to benefit our care provision... we are asked to volume up in case of missing alarms, but can you really keep the phone loud when caring for someone sleeping? |
|  |  | View the system use as a collective event | Accept the inconsistency of technology, and see technology use as a collective event | ...Also we work as team, and I can thus support my colleagues if they have difficulties in using the system...listening to the regulations limit us to provide flexible and high quality care sometimes. |
